# Regional citrate anticoagulation for continuous renal replacement therapy in pediatric patients with liver failure

**DOI:** 10.1371/journal.pone.0182134

**Published:** 2017-08-08

**Authors:** Keila Rodriguez, Poyyapakkam R. Srivaths, Leyat Tal, Mary N Watson, Alyssa A. Riley, Ryan W. Himes, Moreshwar S. Desai, Michael C. Braun, Ayse Akcan Arikan

**Affiliations:** 1 Baylor College of Medicine, Department of Pediatrics, Houston, Texas, United States of America; 2 Baylor College of Medicine, Department of Pediatrics, Renal Section, Houston, Texas, United States of America; 3 Texas Children’s Hospital, Houston, Texas, United States of America; 4 Baylor College of Medicine, Department of Pediatrics, Liver Section, Houston, Texas, United States of America; 5 Baylor College of Medicine, Department of Pediatrics, Section of Critical Care Medicine, Houston, Texas, United States of America; Medizinische Universitat Graz, AUSTRIA

## Abstract

Pediatric liver failure patients frequently develop multiple organ failure and require continuous renal replacement therapy (CRRT) as part of supportive therapy in the pediatric intensive care unit. While many centers employ no anticoagulation for fear of bleeding complications, balanced coagulation disturbance predisposes these patients to clotting as well as bleeding, making maintenance of longer circuit life to deliver adequate dialysis clearance challenging. Regional citrate anticoagulation (RCA) is an attractive option as it avoids systemic anticoagulation, but since citrate metabolism is impaired in liver failure, concerns about toxicity has limited its use. Pediatric data on RCA with liver failure is very scarce. We aimed to establish safety and efficacy of RCA in pediatric liver failure patients on CRRT. Retrospective review of pediatric patients with liver failure receiving CRRT over 30 months. Demographic data and CRRT related data were collected by chart review. Citrate accumulation (CA) was defined as total calcium (mg/dl) /ionized calcium (mmol/L) ratio >2.5 for > 48 hours. Efficacy was assessed by filter life. Safety was assessed by frequency of adverse events ((AEs) defined as bleeding, hemodynamic instability, arrhythmias). Fifty-one patients (median age 3.5 (IQR 0.75–14.2) years) received 861 CRRT days; 70% experienced at least one episode of CA, only 37% were recorded as such in the medical record. AE rate was 93/1000 CRRT days and did not differ between CA days and others. Median filter life was 66 hours (IQR 29–74); 63% filters lasted longer than 48 hrs. Though common, CA was not associated with increased AEs on in pediatric liver failure patients on CRRT receiving RCA. Filter life was adequate. RCA appears an effective anticoagulation for CRRT in pediatric liver failure. Application of a structured definition would increase recognition of CA to allow timely intervention.

## Introduction

Pediatric acute and acute-on-chronic liver failure can lead to multiple organ failure and associated acute kidney injury (AKI) requiring continuous renal replacement therapy (CRRT) [1]. Etiologies for AKI in this setting are often multifactorial and can include exposure to nephrotoxic medications, abdominal compartment physiology, hepatorenal syndrome, and de novo intrinsic renal disease [1]. Despite maximum supportive care, the prognosis for patients with these multi-system disorders carries high morbidity and mortality [2] [3]. A multicenter registry of pediatric CRRT patients reported an overall survival of 58% with lowest survival of 31% observed in liver failure/liver transplant group [3].

Underlying bleeding diatheses resulting from the primary disease process complicates anticoagulation in liver failure patients especially when extracorporeal therapies are used, with serious bleeding complications reported in 16–25% of patients [4,5]. Heparin is the most commonly used anticoagulant for extracorporeal therapies but since it is systemically administered, it carries risks of hemorrhage as well as heparin induced thrombocytopenia leading to a search for other safer options. [6] Regional anticoagulation with citrate (RCA) is the standard of care in our institution for all pediatric patients on CRRT. Citrate works by chelating ionized calcium, a cofactor for multiple steps in the coagulation cascade. The report from pediatric prospective CRRT included 230 circuits with heparin anticoagulation and 158 circuits with citrate anticoagulation; 9 episodes of bleeding were observed with heparin based anticoagulation; however, no mention is made regarding bleeding episodes with citrate anticoagulation [7]. In a study by Rico et al, the bleeding complications observed were 30% in children receiving CRRT, irrespective of heparin or citrate anticoagulation [8]. Citrate is thought to have the benefits of decreasing inflammation as well as the need for blood products and allows longer circuit survival [9–14]. Most of the citrate is cleared by the CRRT circuit; however, about 1 mmol/kg spills over into the patient’s circulation [15,16]. In the setting of liver failure, metabolism of citrate is impaired and accumulation can lead to a citrate toxicity-often known as “citrate lock” -where there is continued chelation of serum ionized calcium [17]. Possible sequelae of the resultant hypocalcemia include arrhythmias, bleeding, and hemodynamic instability [18]. In addition when citrate metabolism is impaired, accumulating citrate can lead to metabolic acidosis in the patient. A total to ionized calcium (mg/mg) ratio that exceeds 2.5 has been reported in the adult literature as a sign of excessive citrate accumulation (CA) [17]. There currently is no consensus definition for CA validated in pediatric patients, which makes recognition and management by physicians challenging [19].

We studied the population of pediatric patients receiving CRRT with concomitant acute and acute-on-chronic liver failure to establish safety and efficacy of RCA in pediatric liver failure. In addition, we applied a standard definition of CA to establish frequency and rate of recognition of the condition.

## Patients and methods

We conducted a retrospective review of electronic medical records for patients with liver failure receiving CRRT over 30 months at Texas Children’s Hospital. Patients were identified from an institutional CRRT database based on International Classification of Diseases (ICD) 9 and 10 codes referring to acute and acute on chronic liver failure by one investigator (KR) and confirmed by chart review. Acute liver failure was defined as coagulopathy and hepatic encephalopathy; acute on chronic liver failure was defined as acute deterioration of a preexisting, chronic liver disease, related to a precipitating event, according to American Association for the Study of Liver Diseases [20,21]. Citrate accumulation (CA) was defined as a ratio of total Calcium to ionized calcium greater than or equal to 2.5 for longer than 48 hours. Duration of CA, timing and nature of interventions implemented were recorded. In addition, demographic data including age, weight, and gender, laboratory parameters, percentage of fluid overload, and use of vasoactive infusions were collected. Primary outcomes evaluated were safety and efficacy related to RCA use, that is, frequency of biochemical evidence of CA, adverse events (AEs) (defined as hemodynamic instability requiring intervention, arrhythmias, and bleeding events), severe metabolic acidosis (defined as pH<7.1), severe metabolic alkalosis (defined as pH>7.5) (as previously reported [22,23]; efficacy was assessed by filter life. Pediatric intensive care unit (PICU) length of stay (LOS), hospital LOS, duration of ventilation, days on CRRT, and mortality were recorded. Baylor College of Medicine Institutional Review Board approved this study and waived requirement for informed consent. All data used in analyses were deidentified and analyzed and reported in aggregate.

As per institutional protocol, all CRRT patients receive hemodiafiltration (CVVHDF) at minimum starting dose of 2000 ml/1.73m2/hour with commercial bicarbonate based dialysate and replacement solutions. CVVHDF was prescribed with prefilter dilution and RCA, filtration fraction was kept below 25%. Commercial calcium free dialysate and replacement fluids were used for CVVHDF. Citrate solution used was anticoagulant citrate dextrose solution A (2.13% citrate). All patients also received calcium chloride (8mg/ml) or calcium gluconate (20mg/ml) infusions either into return line of CRRT circuit or via separate central line. Starting citrate and calcium infusions were prescribed relative to blood flow (Qb; 1 x and 0.6 x Qb/hour, respectively) and titrated according to serum and circuit ionized calcium levels. Postfilter ionized calcium levels were monitored routinely at least every 8 hours and kept between values determined by the attending nephrologist. Institutional protocol targets postfilter ionized calcium of 0.2–0.4, but depending on the severity of liver dysfunction, this interval was changed to 0.5–0.7 at the attending nephrologist’s discretion. Patient ionized calcium levels were also monitored with the same frequency and calcium infusion was adjusted accordingly. In patients less than 15 kg, packed red blood cells diluted with normal saline to a hematocrit of 35% were used to prime the CRRT machine before treatment initiation per the institutional protocol.

Patients received therapeutic plasma exchange (TPE) for refractory coagulopathy (defined as requirement of greater than 30 ml/kg of fresh frozen plasma (FFP) in 24 hours for coagulopathy or clinical bleeding) or thrombocytopenia associated multiple organ failure during CRRT per institutional guidelines. Centrifugal TPE with all FFP replacement and 1–1.5 x plasma volume was performed in tandem with CVVHDF, where centrifugal plasma exchange device was connected in series with CRRT. Patient ionized calcium levels were monitored every 15 minutes during tandem TPE, no separate anticoagulation or additional citrate was used to anticoagulate the plasma exchange circuit but calcium infusion was adjusted following serum ionized calcium levels.

Continuous variables were reported as means +- standard deviation or medians and interquartile ranges and compared using Student’s t test or Mann-Whitney when appropriate. Categorical variables were reported as percentages and compared using chi-square or Fisher’s exact test. All statistical analyses were performed using STATA version 12.0 (College Station, Texas).

## Results and discussion

Fifty-one patients with acute and acute-on-chronic liver failure received a total of 861 days of CRRT during the study period, 36 (71%) were female (Table 1). The median age was 3.5 (IQR 0.75–14.2) years. Eighteen (35%) were infants younger than one year of age. The most common etiology of liver failure was biliary atresia at 37% (n = 19). Hospital mortality was 57% (n = 29). Forty-eight patients (94%) were on at least one vasoactive amine during the PICU stay. Median hospital LOS was 40 (25–97) days, median PICU LOS was 27 (14–55) days, and median duration of ventilation was 17 (7–29) days. Forty one percent (n = 21) of the patients received an orthotopic liver transplant (OLT) during hospital stay; of these 10 were bridged to OLT on CRRT; 11 had primary graft nonfunction or dysfunction or hepatic artery thrombosis and were started on CRRT after OLT. Nine of the patients who were bridged to OLT survived to hospital discharge (90%), whereas only 6/11 (55%) survived in the latter group, two patients in this latter group underwent repeat OLT due to hepatic artery thrombosis. Overall survival to discharge in the first OLT group was 72%.

**Table 1:** Demographic characteristics and clinical outcomes of the cohort.

Parameter	Median (IQR), mean ± SD, or n (%)
Age	3.5 (IQR 0.75–14.2)
Age groups	
< 1 yr	18 (35%)
1–2 years	7 (13.7%)
2–8 years	10 (19.6%)
8 years	16 (31.4%)
Gender, female	36 (71%)
Admission weight, kg	13.8 (9.6–49.8)
Mechanical ventilation, n (%)	49 (96.1%)
Length of mechanical ventilation, days	17 (7–29)
Inotrope use, n(%)	48 (94%)
Length of hospital stay, days	40 (25–97)
Length of PICU stay, days	27 (14–55)
Primary liver disease	30 (61.2%)
Etiology for liver failure	
Biliary atresia	19 (37.3%)
Metabolic	5 (9.8%)
HLH	2 (3.9%0
Shock liver	3 (5.9%)
Hepatitis, NOS	4 (7.8%)
Autoimmune	4 (7.8%)
Multiple organ failure	2 (3.9%)
Other	12 (23.5%)
Peak total bilirubin, mg/dL	22.6±18.7
Peak conjugated bilirubin, mg/dL	18.1±14.4
Nadir albumin, g/dL	2.1 (1.8–2.2)
Peak INR	4.2 (2.6–6.3)
Nadir serum sodium, mEq/L	132 (127–135)
Platelets at CRRT start (10^3^/μL)	81.8 ± 53.6
Total calcium, mmol/L	2.6±0.5
Ionized calcium, mmol/L	1.2±0.2
Peak lactate	6.4 (5.2–12)
Hospital mortality	29 (56.9%)
Orthotopic liver transplant, n (%)	26 (51%)
Evaluated for OLT	35 (68.6%)
Listed for OLT	33/35 (94.3%)
Bridged to OLT on CRRT, n (%)	10 (19.6%)

PICU, pediatric intensive care unit; HLH, hemophagocytic lymphohistiocytosis; NOS, not otherwise specified; INR, international normalized ratio; CRRT, continuous renal replacement therapy; OLT, orthotopic liver transplantation.

The most common indication for initiating CRRT was AKI at 57% (n = 29). Median duration of CRRT was 11 (5–21) days. Mean percentage of overload (FO%) at CRRT initiation was 22.3± 20.1%. Twenty three (45%) of patients had FO% greater than 15% at CRRT initiation. Mean FO% for survivors was 22.3 ± 22.3% and for non-survivors was 22.3 ± 18.7% (p = 0.6).

Thirty five (70%) of the patients experienced at least one episode of CA according to our definition (total Ca/Ionized Ca >2.5 for > 48 hours). Median duration of CA was 3 days (IQR 0–8). Of CA patients, 15 (42.9%) were noted in the electronic medical record as being in “citrate toxicity” by a nephrologist and four additional patients were noted to be “at risk”. Interventions for citrate toxicity included decreasing the dose of citrate, increasing diffusive clearance, adjusting ultrafiltration rate and transiently stopping citrate. Treatment was interrupted in only two patients, for less than six hours each. Although hypocalcemia was common, it was short lived in almost all cases, only 6% (613/9483) of the ionized calcium values were less than 1 mmol/L.

Twenty patients received 5.8 ± 3.8 TPE sessions with all FFP replacement in tandem with CRRT. TPE patients were more likely to have CA than others (18/20 (90%) vs 17/31 (55%), p = 0.004).

There were a total of 79 adverse events (defined as bleeding requiring clinical intervention, hypotension requiring intervention, arrhythmia requiring intervention) in 34 patients; an event rate of 92 events per 1000 CRRT days. Of these, 29 events in 22 patients coincided with days of citrate lock, timing of the other 50 AEs were not associated with dates of citrate lock (p = 0.12). Sixty percent of the patients with CA and 53% of the patients without CA experienced at least one episode of hypotension requiring intervention (p = 0.76) (Table 2). Four out of 14 patients without CA (30%) vs 14/31 (45%) with CA experienced at least one episode of bleeding (p = 0.3). One patient suffered a cardiac arrest secondary to hemorrhagic shock associated with hemodialysis catheter insertion prior to CRRT start, but was successfully resuscitated and bridged to OLT and subsequently liberated from dialysis and discharged home without sequelae, with intact liver and renal function. Two patients who were deemed not to be OLT candidates suffered pulmonary hemorrhage as a terminal event. Two patients developed small cerebral hemorrhages while on CRRT. None of these events occurred on dates of CL. In fact, the two cerebral hemorrhages happened after OLT with a working graft; in both cases patients were severely thrombocytopenic and hypertensive. Only 7% of the AEs coincided with hypocalcemia, while 93% of the AEs happened when ionized calcium levels were within normal limits. Nine patients out of 51 (17.6%) had severe metabolic acidosis, 9/51 (17.6%) had severe metabolic alkalosis, 4 patients had both metabolic acidosis and alkalosis, though not simultaneously. Neither of the acid-base abnormalities were more common in patients with CA. Duration of CA was not associated with hypotension, bleeding, acidosis, or alkalosis events. AEs were more common in the TPE group (1.0 ± 1.1 *vs* 2.3± 2.7, p = 0.02). Only one patient developed cardiac arrhythmia leading to hemodynamic instability, but that patient never developed CA.

**Table 2:** Continuous renal replacement therapy indications, treatment parameters, adverse events, citrate accumulation.

Parameter	Median (IQR), mean ± SD, or n (%)
CRRT indication	
AKI	29, (57%)
Fluid overload	28, (55%)
Hyperammonemia	14, (27%)
Electrolyte disturbance	5, (10%)
Refractory metabolic acidosis	2, (4%)
CRRT duration	11 (5–21) days
% fluid overload at CRRT start	22.3± 20.1%.
Initial CRRT prescription	
Blood flow rate, ml/min	60 (80–150)
Replacement fluid rate, ml/hr	300 (250–600)
Dialysis rate, ml/hr	450 (300–850)
Therapeutic plasma exchange, n (%)	20 (39.2%)
Number of TPE sessions	5 (3–7.5)
Filter life, hours	66 (29–74)
Filters lost to clotting, n (%)	34 (15%)
Adverse event types	
Hemodynamic instability/hypotension	29 (56.9%)
Arrhythmia	1 (1.9%)
Bleeding	18 (39.1%)
Metabolic alkalosis	9 (17.6%)
Metabolic acidosis	9 (17.6%)
Patients with hypocalcemia (ionized calcium < 1.0 mmol/L on any occasion)	49 (96.1%)*
Hypocalcemia episodes, n = 9483**	613 (6%)
Severe hypocalcemia (Ionized calcium < 0.6 mmol/L)	0
Ionized calcium < 0.8 mmol/L^$^	10 (0.1%)
Patients with hypercalcemia (ionized calcium > 1.3 mmol/L on any occasion)	34 (69.4%)
Citrate accumulation, days	3 (0–8)

*In 13 patients, hypocalcemia occurred without evidence of CA. All cases of hypocalcemia responded well to treatment and were not sustained.

^$^ All of these patients were receiving concomitant TPE

** Represents all the ionized calcium values drawn on the patients throughout the CRRT duration

CRRT, continuous renal replacement therapy; AKI, acute kidney injury; TPE, therapeutic plasma exchange

In univariate analyses, only receiving TPE and number of TPE sessions were associated with CA. Duration of CRRT, receiving TPE, number of TPE sessions, and peak conjugated bilirubin were associated with duration of CA; age, peak total bilirubin, peak INR, lowest serum albumin were not (Table 3). Contrary to reports in adults, peak lactate levels were not associated with CA development, even though lactate levels were higher in nonsurvivors [24]. Similarly, CA development did not predict mortality.

**Table 3:** Univariate analysis of parameters associated with citrate accumulation duration.

parameter	coefficient	95% CI	p
age	-0.27	-0.37–0.32	0.8
peak INR	0.19	-0.69–1.06	0.6
peak total bilirubin	0.14	-0.01–0.29	0.07
peak conjugated bilirubin	0.24	0.08–0.40	0.004
lowest serum albumin	6.04	-0.69–12.78	0.07
Duration of CRRT	0.27	0.15–0.39	<0.001
Receiving TPE	6.52	1.68–11.37	0.01
Number of TPE sessions	1.13	0.53–1.73	<0.001

INR, international normalized ratio; CRRT, continuous renal replacement therapy; TPE, therapeutic plasma exchange

In multivariate analyses, only TPE was independently associated with occurrence of CA when controlled for age, peak conjugated bilirubin, peak INR, lowest serum albumin, and duration of CRRT (Table 4). In contrast, duration of CRRT, peak conjugated bilirubin and lowest serum albumin were independently associated with duration of CA when controlled for the same variables. Peak conjugated bilirubin levels were also independently associated with AEs (coefficient = 0.07 (0.01–0.13, p = 0.02).

**Table 4:** Multivariate analyses for citrate accumulation occurrence and duration.

parameter	Coefficient/OR	95% CI	p
CA occurrence	Odds Ratio		
CRRT duration	1.02	0.96–1.09	0.5
TPE	19.87	1.66–237.96	0.02
Age	0.93	0.82–1.03	0.2
Peak conjugated bilirubin	0.98	0.93–1.04	0.5
Peak INR	1.19	0.87–1.63	0.3
Lowest serum albumin	3.16	0.29–34.13	0.3
CA duration	Coefficient		
CRRT duration	0.28	0.16–0.39	<0.001
TPE	2.11	-1.80–6.04	0.3
Age	0.13	-0.12–0.39	0.3
Peak conjugated bilirubin	0.15	0.02–0.28	0.02
Peak INR	0.31	-0.34–0.95	0.3
Lowest serum albumin	9.98	4.79–15.18	<0.001

CA, citrate accumulation; INR, international normalized ratio; CRRT, continuous renal replacement therapy; TPE, therapeutic plasma exchange

Filter life data was available for 223 filters. Two patients had missing filter data. Median filter life was 66 hours (IQR 29–74). Most common reason for filter change was elective. Thirty-four filters (15%) were lost due to clotting. Sixty three percent of filters survived longer than 48 hours and 33% survived longer than 72 hours.

## Discussion

Biochemical evidence of CA was common in pediatric liver failure patients on CRRT and RCA but was not associated with AEs or clinical decompensation. Introducing a standardized definition of CA using total calcium to ionized calcium ratio would increase recognition of CA in the clinical setting and facilitate timely intervention.

Citrate serves as an anticoagulant by chelating serum calcium thereby blocking completion of the coagulation cascade. RCA, first used for patients in which systemic anticoagulation was contraindicated, continues to be used as an alternative to heparin and other anticoagulating agents due to the presumed associated decrease in bleeding episodes and increased safety [25–27]. An otherwise healthy individual receiving CRRT would metabolize citrate hepatically; citrate toxicity becomes an issue in the patient population with concomitant hepatic dysfunction or failure. A majority of our population experienced CA according to the definition we set. Episodes of CA were not always recognized by nephrologists and noted in the patients’ medical records. RCA has been viewed as relatively contraindicated in the liver failure population due to concerns of citrate toxicity. Recent reports have disputed this long-held belief [28–30]. Despite frequent occurrence of CA in our patients, adverse event rate was low with 93 events per 1000 CRRT days. Pediatric liver failure patients requiring CRRT are very sick, evidenced by requirement for mechanical ventilation and vasoactive amine support in almost the entire population. AEs happened on CA days and non-CA days with similar frequency. Hemodynamic and bleeding complications were experienced throughout the courses of CRRT but occurrence was not linked to dates of CA. This can be explained by the high incidence of cardiovascular failure and coagulopathy related bleeding that is a direct result of underlying severe liver failure rather than added complications from RCA. CRRT duration seemed to be negatively associated with CA; we think this is related to survival bias, since the patients with sicker livers will die thereby patients who survive are able to metabolize citrate better.

TPE was associated with increased frequency of CA. Even though no additional citrate was used during TPE sessions; exchange was done with 100% FFP which has a high content of citrate as citrate is the anticoagulant of choice in banked blood products. The added citrate load from the FFP might explain the increased occurrence of CA in TPE patients. Careful monitoring of patient ionized calcium is required during TPE treatments in liver failure and TPE should always be done in tandem with CRRT to facilitate citrate clearance. Another explanation is the possibility that more patients with acute on chronic liver failure received TPE as peak conjugated bilirubin was associated with TPE treatments and might be a marker for more chronic liver disease. However, there was no difference in peak conjugated bilirubin levels in patients who received TPE versus those who did not, nor was there a relationship between underlying liver disease and whether or not TPE was done. In this case, peak conjugated bilirubin might be a surrogate marker of severity of liver disease; a similar explanation might be used for lowest albumin concentrations—which are also markers of chronic disease- and were also independently associated with CA duration. However, we did not demonstrate a relationship between peak INR levels and CA. Peak conjugated bilirubin levels were associated with a shorter CA duration, possibly selecting for patients who succumbed to underlying liver failure and had shorter citrate exposure.

We investigated the occurrence of metabolic acidosis, since citrate load, in the setting of impaired metabolism, will lead to metabolic acidosis as previously reported [24,25]. We elected to use similar cutoffs to previously published [23,24]. Patients experienced numerous episodes of acid-base abnormalities, mostly due their underlying disease or comorbidities, as a great majority had respiratory failure with varying degrees of lung disease as well as shock and/or sepsis; we elected to focus on isolated and severe metabolic acidosis and alkalosis events. Episodes of isolated metabolic acidosis and alkalosis were infrequent and not related to CA days. Prolonged CRRT treatment could lead to higher citrate load and result in metabolic alkalosis over time. Surprisingly, duration of CRRT was not associated either with metabolic alkalosis or acidosis. Duration of CRRT; however, was associated with CA duration.

RCA was effective as an anticoagulant as evidenced by the long filter lives and low incidence of filter clotting in our patients. Deep et al reported average filter lives of just over 30 hours in pediatric patients with liver failure receiving CRRT when heparin or prostacyclin anticoagulation was used [31]. Longer filter lives and fewer incidences of clotting have been reported in other pediatric patients with RCA compared to heparin [7,9,32,33]. Here, we report a similar finding in liver failure patients. The acceptable adverse event profile, while still requiring close monitoring, make RCA a superior option in terms of adequate treatment delivery by preventing frequent filter changes.

Limitations of our study include the retrospective nature of data collection. AEs were determined through screening of medical records and, as no standardized definitions existed during patient care, are subject to observer and reporter bias due to the lack of a uniform case report form. In order to maximize capturing of all AEs, all notes including the nursing notes were reviewed by a single investigator to provide consistency. Single center data is difficult to extrapolate to other centers as it is impacted by institutional practices. However, RCA is being used increasingly more commonly in pediatric CRRT populations and we propose our patients would be similar to the kinds of patients cared for in other tertiary and quaternary pediatric liver transplant centers.

## Conclusion

Anticoagulation and CRRT in pediatric patients who have liver failure can be a challenging balancing act. Adverse events were common, similar to liver failure patients who are not on extracorporeal therapy; majority happened when ionized calcium levels were in the normal range. Early recognition of CA may be facilitated with the use of standard criteria improving medical management and preventing treatment interruption. TPE use in pediatric patients with liver failure requires close attention to citrate accumulation, especially when done simultaneously with RCA.
